# Supplementary material for: Spatiotemporal control of coacervate formation within liposomes
Source: Nat Commun. 2019 Apr 17;10:1800. doi: 10.1038/s41467-019-09855-x (PMC6470218; doi:10.1038/s41467-019-09855-x)
Supplement: Supplementary file 1 — Supplementary Information [file 41467_2019_9855_MOESM1_ESM.pdf]

SUPPLEMENTARY INFORMATION

Spatiotemporal control of coacervate formation within liposomes

Deshpande et al.

Supplementary Figures

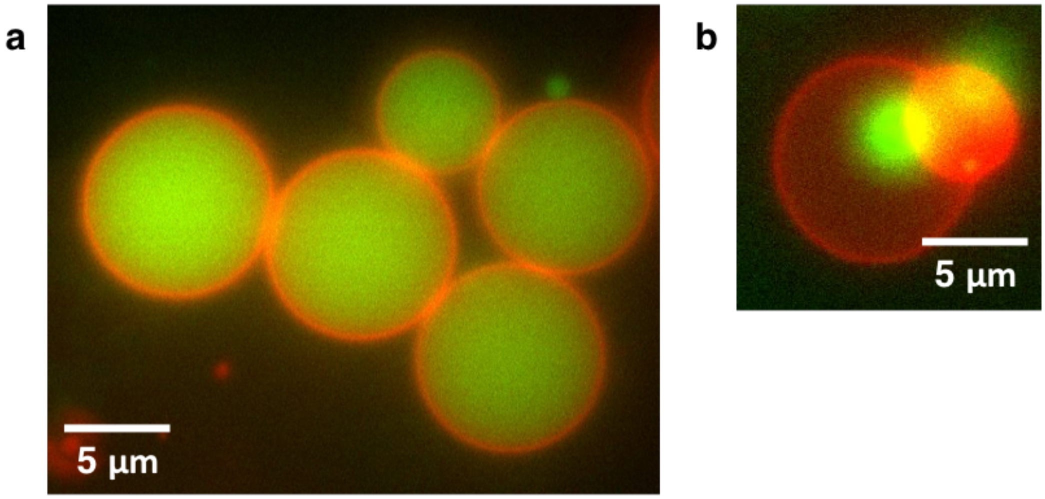

Supplementary Figure 1: Coacervation is not observed inside the liposomes in absence of α-hemolysin pores. (**a**) Liposomes (red) encapsulating pLL polymers (green) do not form coacervates in absence of membrane pores, as ATP molecules cannot transport across the membrane. The image is taken at least 20 minutes after the liposome was in contact with the ATP solution [10 mM]. (**b**) Interestingly, liposomes that had a 1-octanol pocket still attached to them did develop coacervates, possibly due to the transport of ATP through the 1-octanol-lipid interface.

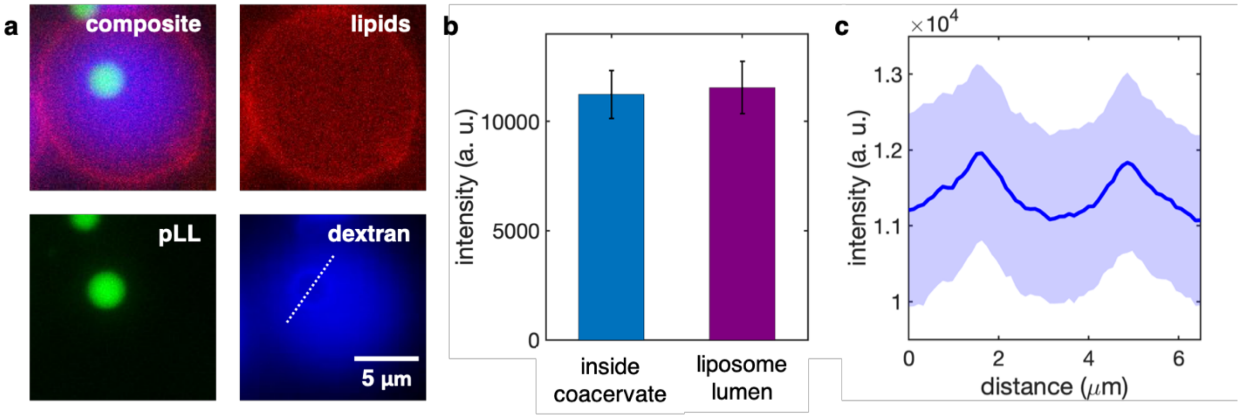

Supplementary Figure 2: Dextran does not play a role in the coacervation process. (**a**) A composite fluorescence image (upper left subpanel) shows that fluorescent dextran (AF647-dextran, shown in blue) is not accumulated inside the coacervate (green). Upon closer inspection, there seems to be a slight (9% more intensity) accumulation of dextran just outside the surface of coacervates. Individual fluorescence channels are separately shown. (**b**) Average fluorescence intensity of AF647-dextran inside the coacervates and in the liposome lumen shows similar values (n = 13). Error bars indicate standard deviations. (**c**) An average line profile across the coacervate, as indicated by a dotted line in the dextran channel in panel a, shows slight accumulation of dextran molecules at the coacervate interface (n = 13). The shaded region indicates the standard deviation.

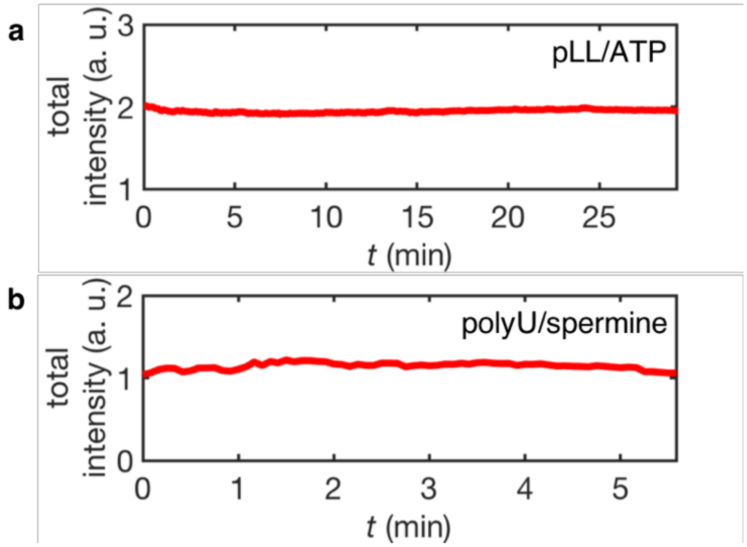

Supplementary Figure 3: No photobleaching is observed while measuring the coacervation dynamics. The total fluorescence intensity of a liposome that did not undergo coacervation stayed constant over a prolonged period of time for both the coacervation systems that were studied. We thus conclude that the fluorescence intensity analyses were not affected by photobleaching.

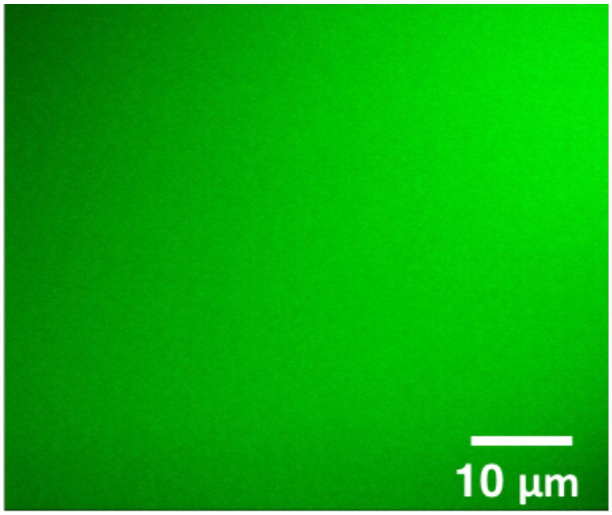

Supplementary Figure 4: pLL and ADP do not form coacervates. A solution containing 5 mg/mL pLL, 0.25 mg/mL cy5-pLL, 10 mM ADP, 150 mM KCl, 5 mM $MgCl_2$, and 25 mM Tris-Cl (pH 7.4) does not phase separate and shows a homogenous fluorescence of cy5-pLL, indicating the complete absence of coacervation.

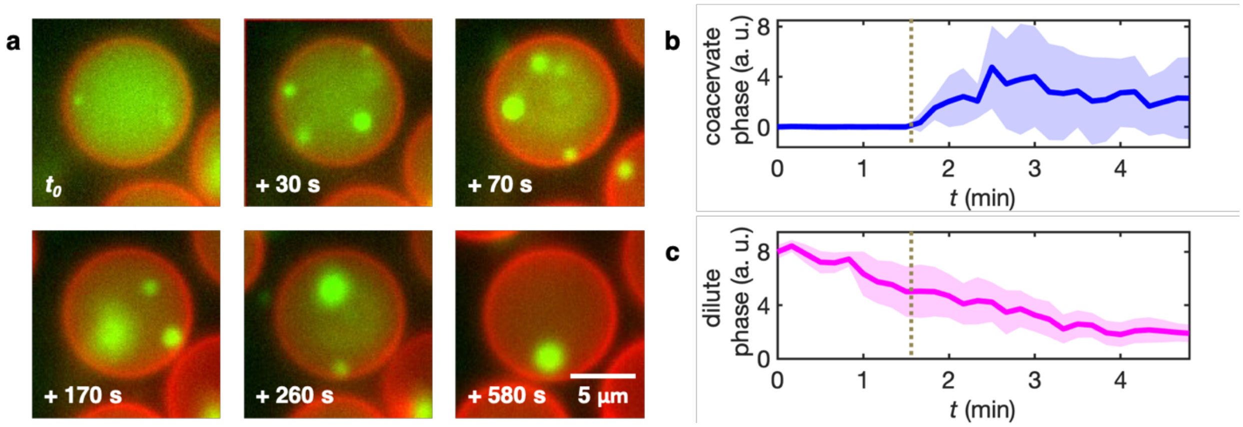

Supplementary Figure 5: pLL and ATP undergo a similar coacervation process in the absence of apyrase. (**a**) Time-lapse fluorescence images showing coacervate formation as ATP molecules diffuse across the membrane to coacervate with pLL polymers. The process looks very similar to when apyrase is present inside the liposome, indicating that apyrase does not affect the induction of coacervation. (**b**) A plot showing the coacervate-phase counts over time (n = 11). The rapid transition from a homogeneous solution to a condensed phase leads to sudden rise in the fluorescence intensity, which plateaus over time. (**c**) Plot of the dilute-phase counts over time (n = 11), showing a complementary rapid decay. The dashed vertical line indicates the onset of coacervation. Lines are the average values and the shaded regions indicate standard deviations. Source data are provided as a Source Data file.

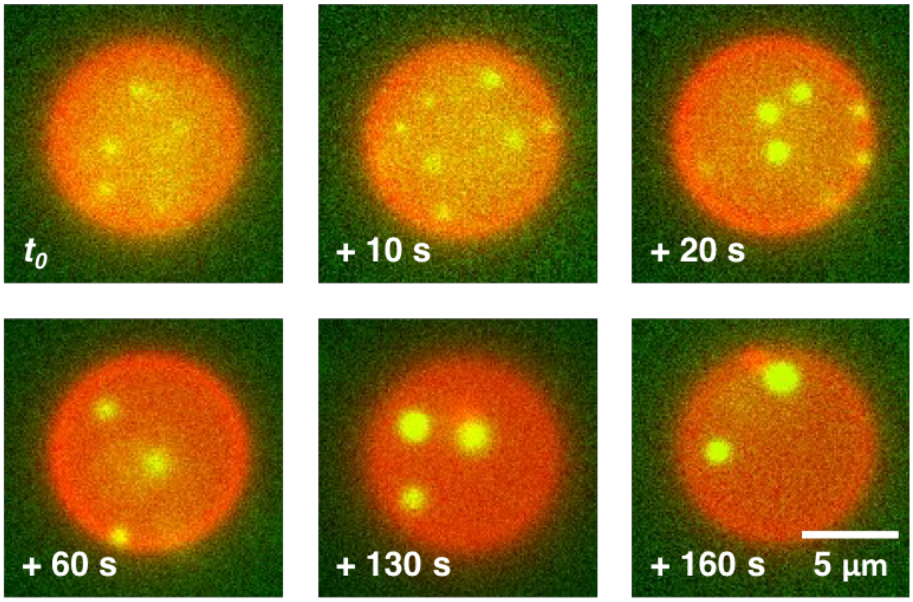

Supplementary Figure 6: pLL and ATP undergo a similar coacervation process in leaky liposomes. Time-lapse fluorescence images showing the coacervation process happening inside a (rare) leaky liposome, in absence of membrane pores. The process, including the onset of coacervation, looks very similar to the one observed in porous liposomes.

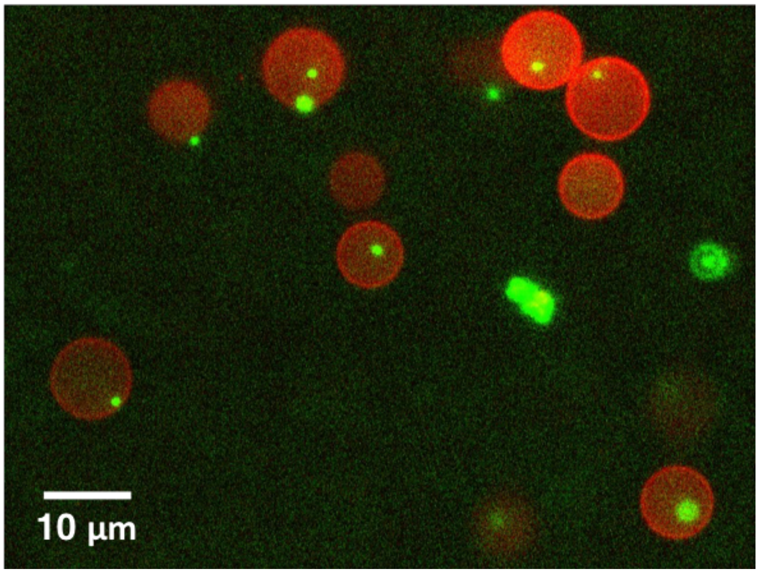

Supplementary Figure 7: PolyU/spermine coacervates remain stable and do not increase in size over the course of hours. Fluorescence image showing several liposomes with polyU/spermine coacervates inside them, more than 2 hours after the coacervate induction. The coacervate size is very much comparable to what is observed within first 10 minutes after coacervation starts.

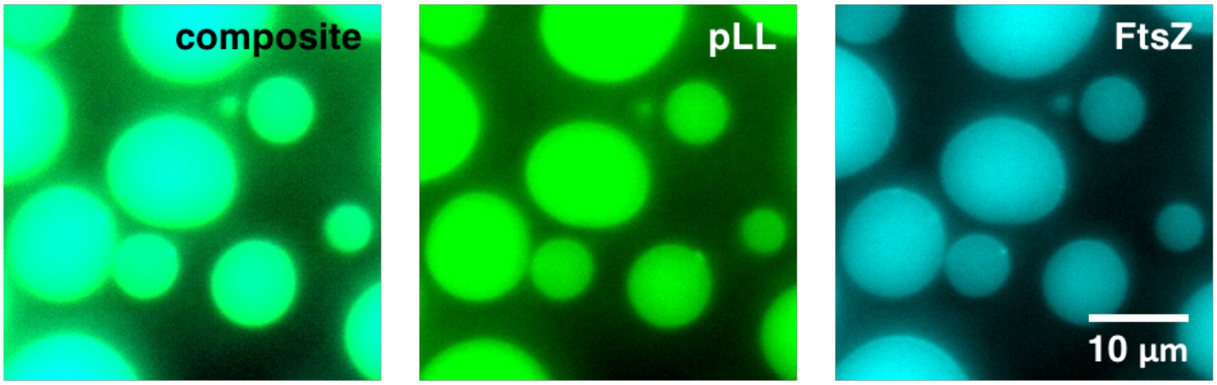

Supplementary Figure 8: In absence of liposomal confinement, FtsZ is partitioned inside pLL/ATP coacervates and shows a homogenous distribution. Set of fluorescence images showing that FtsZ (cyan) is evenly distributed inside the coacervates (green). The coacervates were formed in bulk and visualized on a glass slide. The solution contained 5 mg/mL pLL, 0.25 mg/mL cy5-pLL, 10 mM ATP, 5 mM dextran, 150 mM KCl, 5 mM $MgCl_2$, 25 mM Tris-Cl (pH 7.4), and 10 μM FtsZ.

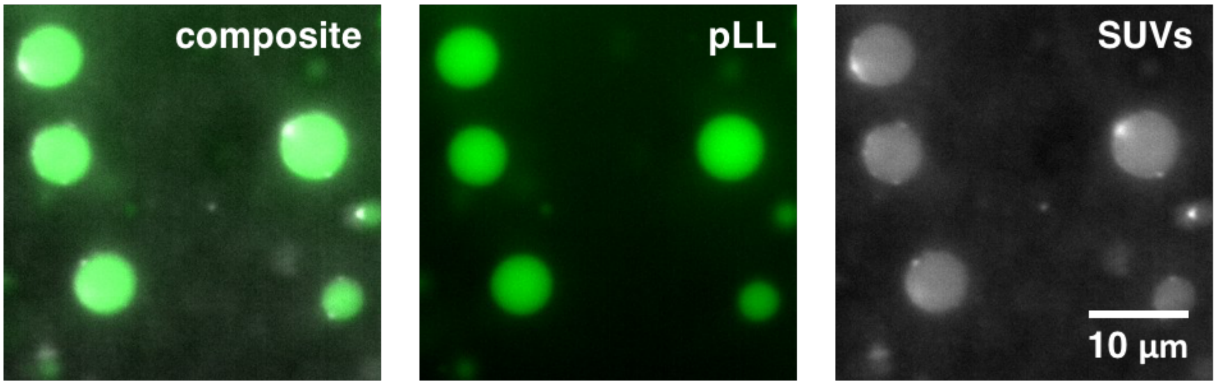

Supplementary Figure 9: In absence of liposomal confinement, SUVs partition inside pLL/ATP coacervates. A set of fluorescence images showing that SUVs (grey) are evenly distributed inside the coacervates (green). The coacervates were formed in bulk and visualized on a glass slide. The solution contained 5 mg/mL pLL, 0.25 mg/mL cy5-pLL, 10 mM ATP, 5 mM dextran, 150 mM KCl, 5 mM $MgCl_2$, 25 mM Tris-Cl (pH 7.4), and 3.5 mg/mL SUVs.

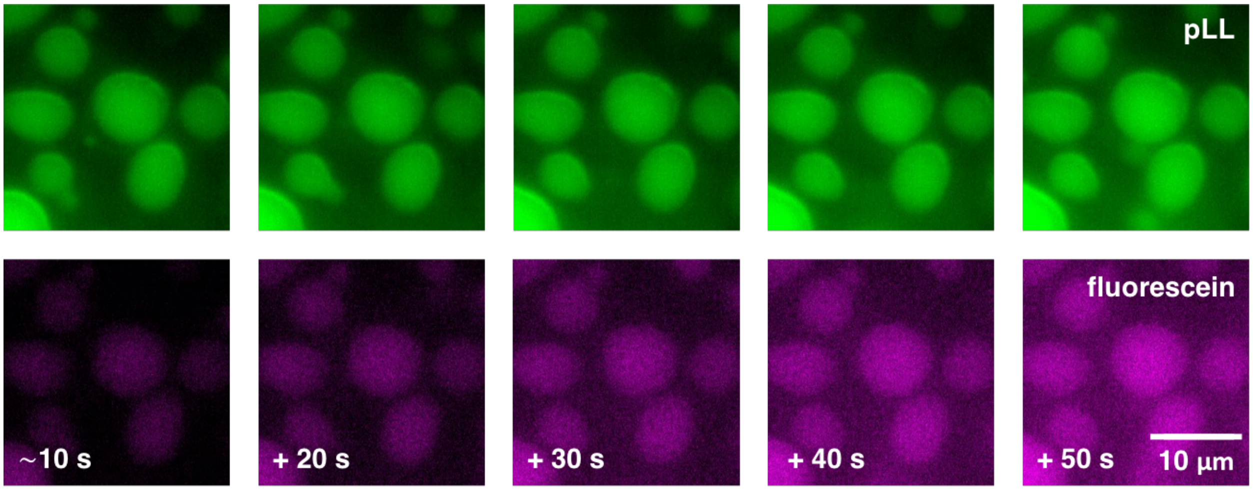

Supplementary Figure 10: β-galactosidase partitions inside pLL/ATP coacervates. Time-lapse fluorescence images showing that the enzymatic reaction is carried out predominantly inside the coacervates, releasing fluorescein, a fluorescent product. The coacervates were formed in bulk and visualized on a glass slide. The solution contained 5 mg/mL pLL, 0.25 mg/mL cy5-pLL, 10 mM ATP, 5 mM dextran, 150 mM KCl, 5 mM $MgCl_2$, 25 mM Tris-Cl (pH 7.4), and 300 units/mL β-galactosidase. A non-fluorescent substrate (FDG) was added at a final concentration of 210 µM. If β-galactosidase would not have partitioned inside the coacervates, we would have observed the fluorescence throughout the dilute phase, before possibly getting concentrated in the condensed phase. The fact that we do not observe this strongly suggests that the enzyme is preferentially partitioned into the coacervates.
