# Supplementary material for: Spatiotemporal control of coacervate formation within liposomes
Source: Nat Commun. 2019 Apr 17;10:1800. doi: 10.1038/s41467-019-09855-x (PMC6470218; doi:10.1038/s41467-019-09855-x)
Supplement: Supplementary file 7 — Description of Additional Supplementary Files [file 41467_2019_9855_MOESM7_ESM.pdf]

Title: Supplementary Movie 1: pLL/ATP coacervate formation inside a liposome.
Description: A typical event showing coacervate formation, as the ATP molecules diffuse through α-hemolysin pores in the membrane (red) and coacervate with the pLL polymers (green) that are encapsulated within the liposome.

Title: Supplementary Movie 2: pLL/ATP coacervate dissolves in presence of apyrase and no additional ATP.
Description: pLL/ATP coacervates adhered to a PDMS-coated substrate dissolve within minutes when a solution containing the enzyme apyrase is flown over them.

Title: Supplementary Movie 3: pLL/ATP coacervate formed inside the liposome is stable.
Description: The formed pLL/ATP coacervate is stable and diffuses freely within the liposome. It continuously exchanges its components with the surroundings as the presence of apyrase does not dissolve it.

Title: Supplementary Movie 4: polyU/spermine coacervate formation inside a liposome.
Description: A typical event showing coacervate formation, as the UDP molecules diffuse through α-hemolysin pores embedded in the membrane (red) and get utilized by PNPase to form polyU polymers (green), which coacervate with spermine molecules present inside the liposome.

Title: Supplementary Movie 5: polyU/spermine coacervate formed inside the liposome is stable.
Description: The formed polyU/spermine coacervate is stable and diffuses freely within the liposome.
